# GTF2E2 is a novel biomarker for recurrence after surgery and promotes progression of esophageal squamous cell carcinoma via miR-139-5p/GTF2E2/FUS axis

**DOI:** 10.1038/s41388-021-02122-8

**Published:** 2021-12-02

**Authors:** Yujie Zhang, Yuxin Zhang, Bo Ai, Juejun Gong, Yichen Li, Shiying Yu, Xiuyu Cai, Li Zhang

**Affiliations:** 1 Department of Oncology, Tongji Medical College, Tongji Hospital, Huazhong University of Science and Technology, No. 1095 Jiefang Avenue, Wuhan, 430030 Hubei China; 2 Hepatic Surgery Center, Tongji Medical College, Tongji Hospital, Huazhong University of Science and Technology, No. 1095 Jiefang Avenue, Wuhan, 430030 Hubei China; 3 Thoracic Surgery Center, Tongji Medical College, Tongji Hospital, Huazhong University of Science and Technology, No. 1095 Jiefang Avenue, Wuhan, 430030 Hubei China; 4 Department of VIP Inpatient, Sun Yet-Sen University Cancer Center, State Key Laboratory of Oncology in South China, Collaborative Innovation Center for Cancer Medicine, No.651 Dongfeng Road East, Guangzhou, Guangdong 510060 P. R. China

**Keywords:** Tumour biomarkers, Prognostic markers

## Abstract

Esophageal squamous cell carcinoma (ESCC) is one of the most lethal gastrointestinal malignancies with high mortality. Recurrence develops within only a few years after curative resection and perioperative adjuvant therapy in 30–50% of these patients. Therefore, it is essential to identify postoperative recurrence biomarkers to facilitate selecting the following surveillance and therapeutic strategies. The general transcription factor IIE subunit beta (GTF2E2) is crucial for physiological and pathological functions, but its roles in the aggression and recurrence of ESCC remain ambiguous. In this study, we found that GTF2E2 was highly expressed in ESCC samples, and elevated GTF2E2 expression predicted early recurrence after surgery for ESCC patients. High expression of GTF2E2 associated with more aggressive clinic features and poor prognosis. GTF2E2 promoted the proliferation and mobility of ESCC cells in vitro and in vivo. We further revealed that miR-139-5p repressed GTF2E2 expression by downregulating its mRNA through binding with Argonaute 2 (Ago2). Rescue assays suggested that miR-139-5p affected GTF2E2-mediated ESCC progression. Moreover, GTF2E2 positively interacted with FUS promoter and regulated FUS expression, and the phenotype changes caused by GTF2E2 manipulation were recovered by rescuing FUS expression in ESCC cells. Additionally, we demonstrated that GTF2E2 promotes ESCC cells progression via activation of the AKT/ERK/mTOR pathway. In conclusion, GTF2E2 may serve as a novel biomarker for recurrence after surgery and a potential therapeutic target for ESCC patients, and it promotes ESCC progression via miR-139-5p/GTF2E2/FUS axis.

## Introduction

Esophageal squamous cell carcinoma (ESCC) is one of the most lethal gastrointestinal malignancies and is the primary cause of cancer-related death in China [1–3]. Recent developments in multimodal treatment have improved the prognosis of patients with esophageal cancer, and surgery is still the mainstay treatment for potentially curable esophageal cancer [4–7]. However, the overall 5-year survival rate is generally only 25–50% [8], and recurrence develops within only a few years after curative resection and perioperative adjuvant therapy in 30–50% of these patients [9–12]. Therefore, it is essential to find promising biomarkers for early detection of recurrence to facilitate selection of the following surveillance and therapeutic strategies in time.

Several general transcription factors assemble into a preinitiation complex (PIC) to ensure accurate RNA pol II loading at the transcription start site. Among them, the general transcription factor IIE subunit beta, also known as GTF2E2, is a crucial component for PIC assembly and stabilization required for transcription initiation and promoter opening by facilitating loading and stable binding of TFIIH [13, 14]. Previous bioinformatical analysis suggested that GTF2E2 regulates the progression of glioblastoma by upregulating the level of the cell division cycle 20 (CDC20) [15]. In addition, GTF2E2 mutation is correlated with remarkable DNA repair-independent transcription defects and tissue-specific dysfunction [16]. Recent research reported that GTF2E2 plays an oncogenic role in lung adenocarcinoma by interacting with RPS4X [17]. These studies demonstrated that GTF2E2 is involved in carcinogenesis. However, the biological function and the molecular mechanism of GTF2E2 in the aggression and treatment failure of ESCC remain ambiguous.

MicroRNAs (miRNAs) are a class of non-coding RNAs of 19–25 nucleotides in length that regulate post-transcriptional gene expression [18, 19]. Instead of directly silencing targeted mRNA, miRNAs function by guiding Argonaute2 (Ago2), the heart of RNA-induced silencing complex (RISC), to complementary sites in target mRNAs to promote mRNA decay or repress mRNA translation [20, 21]. Liu et al. [22] reported that miR-139-5p was correlated to a proliferation- and metastasis-suppressing function in human ESCCs by directly targeting NR5A2. Wen et al. [23] revealed that miR-139-5p was a prognostic prediction factor for overall survival (OS) in LN-positive locoregional ESCC patients. Jiao et al. [24] found that miR-139-5p inhibited the development of esophageal cancer by regulating VEGFR and downstream signaling pathways. Lower miR-139-5p expression in ESCC tissues [25] and esophageal cancer samples [26] was observed compared to that in adjacent non-tumor tissues.

Fused in sarcoma/translocated in liposarcoma (FUS), a nuclear RNA-binding protein, plays an essential role in gene expression, including transcription, splicing, and even mRNA transport [27]. FUS has been reported as an oncogene in various human cancers [28–30]. However, the role that FUS plays in ESCC remains unclear.

In the present study, we demonstrate that GTF2E2 is significantly upregulated in ESCC and markedly correlated with tumor recurrence after surgery of ESCC. Furthermore, we systematically examined GTF2E2’s potential functions in tumorigenesis and metastasis of ESCC. In addition, our study found that miR-139-5p is an upstream regulator of GTF2E2 by decaying GTF2E2 mRNA in an Ago2-dependent manner. miR-139-5p inhibits the proliferation and mobility of ESCC cells by directly regulating GTF2E2. Interestingly, according to the RNA-seq and ChIP-seq results, GTF2E2 was explored as the direct regulator for the transcription of FUS, which was confirmed by ChIP-PCR and dual-luciferase reporter assays. Rescue assays were conducted to illustrate the role of FUS in GTF2E2-mediated ESCC progression. Collectively, our results indicate that GTF2E2 may serve as a novel recurrence biomarker and potential therapeutic target for ESCC.

## Results

### GTF2E2 is upregulated in ESCC samples and cells, and its high expression predicts early postoperative recurrence

**Fig. 1:**
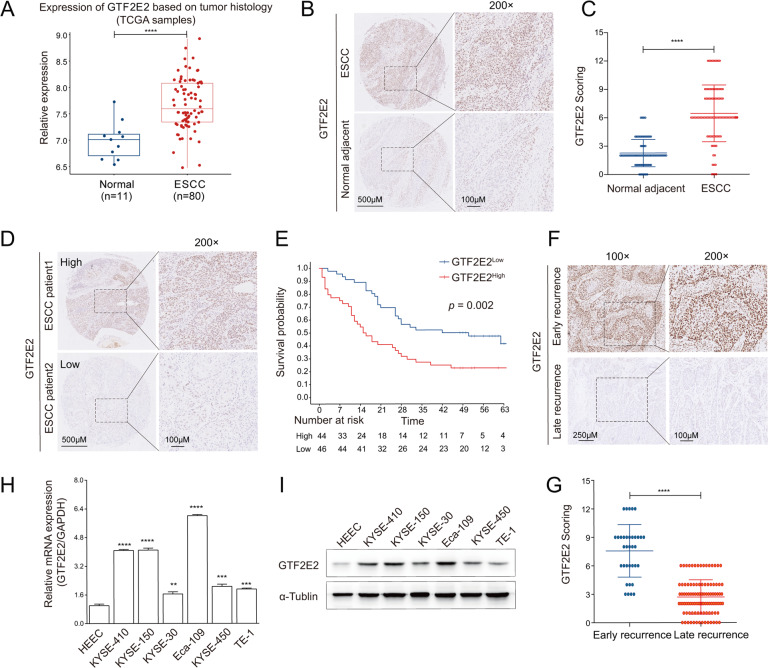
GTF2E2 is upregulated in ESCC samples and cells, and its high expression predicts early postoperative recurrence. A Comparison of the GTF2E2 mRNA level between ESCC and normal tissues in TCGA database. **B**, **C** Representative images (**B**) of IHC staining and the relative IHC scores (**C**) of GTF2E2 in ESCC tissues (*n* = 90) and adjacent normal tissues (*n* = 90). **D**, **E** Representative images of IHC (**D**) and Kaplan–Meier analysis of overall survival probability (**E**) in ESCC patients by GTF2E2 levels. **F**, **G** Representative images (**F**) of IHC staining and the relative IHC scores (**G**) of GTF2E2 in early (*n* = 35) and late (*n* = 106) postoperative recurrent ESCC samples. **H**, **I** The relative expression levels of GTF2E2 measured by RT-PCR (**H**) and western blot (**I**) in six ESCC cell lines and a normal esophageal epithelial cell line, HEEC. GAPDH and α-tublin were used as loading controls, respectively. *p < 0.05, **p < 0.01, ***p < 0.001, ****p < 0.0001 vs. control. *n* = 3.

To reveal the potential biological function of GTF2E2 in ESCC, we explored its expression level in tumor and adjacent normal tissues through bioinformatical and experimental approaches. Based on the TCGA database, the expression of GTF2E2 was shown to be significantly higher in ESCC tumor samples relative to normal tissues (Fig. 1A). The OS was compared between GTF2E2 high (*n* = 39) and low expression (*n* = 40) in ESCC patients from TCGA cohort (Fig. S1A). Next, we performed IHC staining in tissue microarray and found that GTF2E2 expression was elevated in ESCC tissues compared with adjacent counterparts (Fig. 1B, C). The ESCC tissue samples were divided into two groups (high and low) using the median of all scored tumor tissues as the cutoff value (Fig. 1D). As shown in Fig. 1E, high GTF2E2 expression is an effective predictor for poor OS. In addition, it was found that high GTF2E2 expression was correlated with high advanced N stage and clinical stage (Table 1). Moreover, the univariate Cox regression model revealed that N stage, clinical stage, and GTF2E2 expression were associated with the survival of ESCC patients. The multivariate analysis indicated that GTF2E2 expression is an independent prognostic factor for OS in ESCC patients (Table 2). Altogether, these data showed that GTF2E2 is highly expressed in ESCC tissues, suggesting the potential oncogenic activity of GTF2E2 in ESCC. Moreover, high GTF2E2 expression level in ESCC patients is a predictor of poor clinical outcome.

**Table 1:** Correlation of GTF2E2 expression with clinicopathological factors.

		GTF2E2		
Characteristics	*N*	Low (*N* = 46)	High (*N* = 44)	*P* value
**Age (years)**				1.000
≤65	44	22	22	
>65	46	24	22	
**Gender**				1.000
Male	73	37	36	
Female	17	9	8	
**Tumor size (cm)**				0.300
≤4	42	24	18	
>4	48	22	26	
**Tumor location**				0.879
Upper	17	8	9	
Middle	36	18	18	
Lower	37	20	17	
**Pathlogical grade**			0.454	
G1	40	23	17	
G2	43	19	24	
G3	7	4	3	
**T stage**				0.490
T1/T2	16	15	11	
T3/T4	64	31	33	
**N stage**				0.006
N0	45	30	15	
N1–3	45	16	29	
**Clinical Stage**				
I + II	51	33	18	0.005
III + IV	39	13	26	

Median expression level was used as a cutoff to divide the 90 patients into GTF2E2 high group (*n* = 44) and GTF2E2 low group (*n* = 46). Chi-square test. *P* value < 0.05 is significant.

**Table 2:** Univariate and multivariate Cox regression analyses of potential factors for survival in ESCC patients’ survival in ESCC patients.

Variables	Univariate analysis		Multivariate analysis	
	HR (95%)	*P* value	HR (95%)	*P* value
Age (years)	1.412 (0.845–2.360)	0.188		
Gender	1.643 (0.778–3.469)	0.193		
Tumor size (cm)	1.044 (0.624–1.747)	0.869		
Pathological grade (G1 vs. G2–3)	1.490 (0.885–2.509)	0.134		
T stage	0.902 (0.518–1.570)	0.715		
N stage	2.185 (1.297–3.679)	0.003	0.964 (0.290–3.207)	0.953
Clinical stage	2.432 (1.449–4.082)	0.001	2.153 (0.656–7.070)	0.206
GTF2E2 High vs. Low	2.199 (1.308–3.696)	0.003	1.845 (1.077–3.160)	0.026

*P* value < 0.05 is significant.

To further confirm the correlation between GTF2E2 expression and postoperative recurrence in ESCC, IHC staining of GTF2E2 was performed on paraffin sections of human postoperative recurrent ESCC samples. As shown in Fig. 1F, G, high GTF2E2 expression was positively correlated with early postoperative recurrence of ESCC, indicating that GTF2E2 could predict postoperative recurrence in ESCC patients. The clinical characteristics and GTF2E2 IHC staining scores of postoperative recurrence patients are provided in Table S2.

Then, we investigated the expression levels of GTF2E2 in several ESCC cell lines with RT-PCR and western blot analysis normalized to the normal esophageal epithelial cell line, HEEC (Fig. 1H-I), and the results indicated that GTF2E2 was overexpressed in our examined ESCC cell lines.

Taken together, these findings suggest that GTF2E2 may act as an essential indicator for postoperative recurrence in ESCC, thus providing a promising therapeutic and prediction target for cancer prevention.

### GTF2E2 promotes proliferation, migration and invasion of ESCC cells

**Fig. 2:**
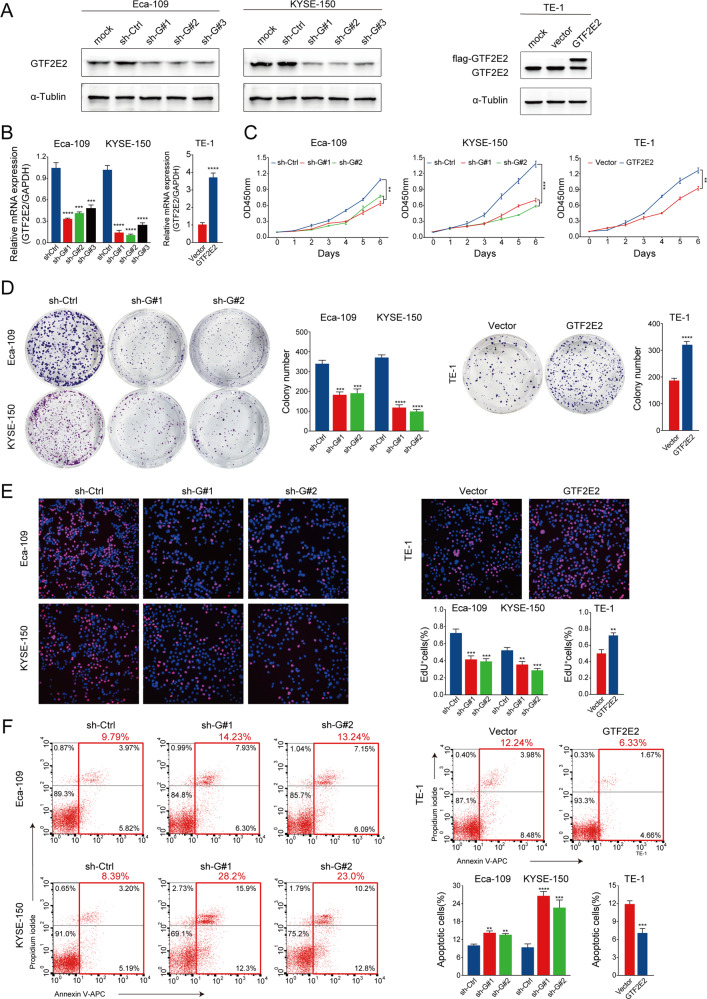
The effects of GTF2E2 on proliferation and apoptosis in ESCC cells. **A**, **B** Western blot (**A**) and RT-PCR (**B**) analysis of GTF2E2 knockdown or overexpression efficiency in the indicated cells. α-Tublin and GAPDH were used as loading controls, respectively. **C**–**E** Overexpression of GTF2E2 promoted proliferation of TE-1 cells in CCK8, colony formation, and EdU assays, while decreased GTF2E2 expression inhibited Eca-109 and KYSE-150 cells progression in the above assays. Quantification of colony numbers and percentages of Edu+ cells. **F** In flow cytometry assay, GTF2E2 overexpression reduced cell apoptosis in TE-1 cells, and downregulation of GTF2E2 increased apoptosis in Eca-109 and KYSE-150 cells. Quantification of percentages of apoptotic cells. *p < 0.05, **p < 0.01, ***p < 0.001, ****p < 0.0001 vs. control. *n* = 3.

The endogenous GTF2E2 expression was found to be relatively low in KYSE-30, KYSE-450, and TE-1 cells, and relatively high in KYSE-410, KYSE-150, and Eca-109 cells (Fig. 1H, I). GTF2E2 was successfully knocked down in Eca-109 and KYSE-150 cells by lentivirus transduction with three independent shRNAs, and GTF2E2 was overexpressed in TE-1 cells (Fig. 2A, B). CCK8, colony formation, EdU, and flow cytometry assays showed that ESCC cells with higher GTF2E2 expression exhibited stronger proliferation ability and reduced apoptosis compared with counterpart cells of lower GTF2E2 expression (Fig. 2C–F).

**Fig. 3:**
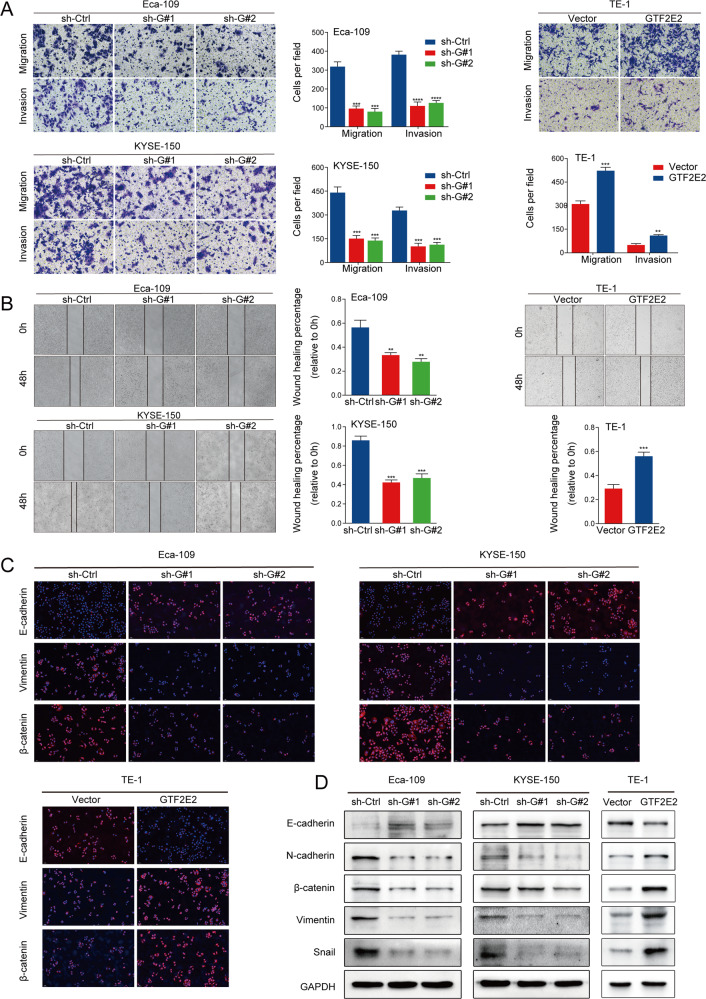
GTF2E2 promotes migration, invasion, and EMT of ESCC cells. **A** Representative images of transwell migration and invasion assay in the GTF2E2-overexpressing TE-1 cells or GTF2E2-knockdown Eca-109 and KYSE-150 cells. Quantification of migrated and invaded cells. **B** In the wound-healing assay, TE-1 cells with GTF2E2 overexpression displayed significantly accelerated wound healing compared with the control, while Eca-109 and KYSE-150 cells with decreased GTF2E2 showed slowed wound healing compared with control cells. Quantification of wound healing percentage. **C** In the immunofluorescence assay, TE-1 cells with increased GTF2E2, and Eca-109 and KYSE-150 cells with decreased GTF2E2 were detected the EMT markers using the corresponding antibodies, as presented in red; DAPI, as shown in blue **D**. In western blot analysis, overexpression of GTF2E2 reduced the expression of epithelial protein maker (E-cadherin) and increased the expression of mesenchymal protein markers (N-cadherin, β-catenin, Vimentin, and Snail) in TE-1 cells, and the reverse trend was observed in Eca-109 and KYSE-150 cells with GTF2E2 downregulation. *p < 0.05, **p < 0.01, ***p < 0.001, ****p < 0.0001 vs. control. *n* = 3.

Transwell and wound-healing assays indicated that overexpression of GTF2E2 promoted and knockdown of GTF2E2 inhibited migration and invasion in ESCC cells (Fig. 3A, B). Furthermore, the western blot results suggested that inhibition of GTF2E2 indeed downregulated the level of mesenchymal markers including N-cadherin, β-catenin, Snail, and Vimentin, and upregulated the level of epithelial marker E-cadherin in ESCC cells. Meanwhile, the reverse trend was shown in ESCC cells in which GTF2E2 expression was increased (Fig. 3D). To directly visualize the epithelial–mesenchymal transition (EMT), we conducted immunofluorescence assay on EMT markers in ESCC cells. In Eca-109 and KYSE-150 cells with inhibition of GTF2E2, the levels of β-catenin and Vimentin decreased and the E-cadherin expression level was elevated, and the reverse phenomenon was displayed in GTF2E2-overexpressed TE-1 cells (Fig. 3C). These suggested that GTF2E2 might induce EMT and promote migration and invasion of ESCC cells.

### GTF2E2 is required for tumorigenesis and metastasis of ESCC cells in vivo

**Fig. 4:**
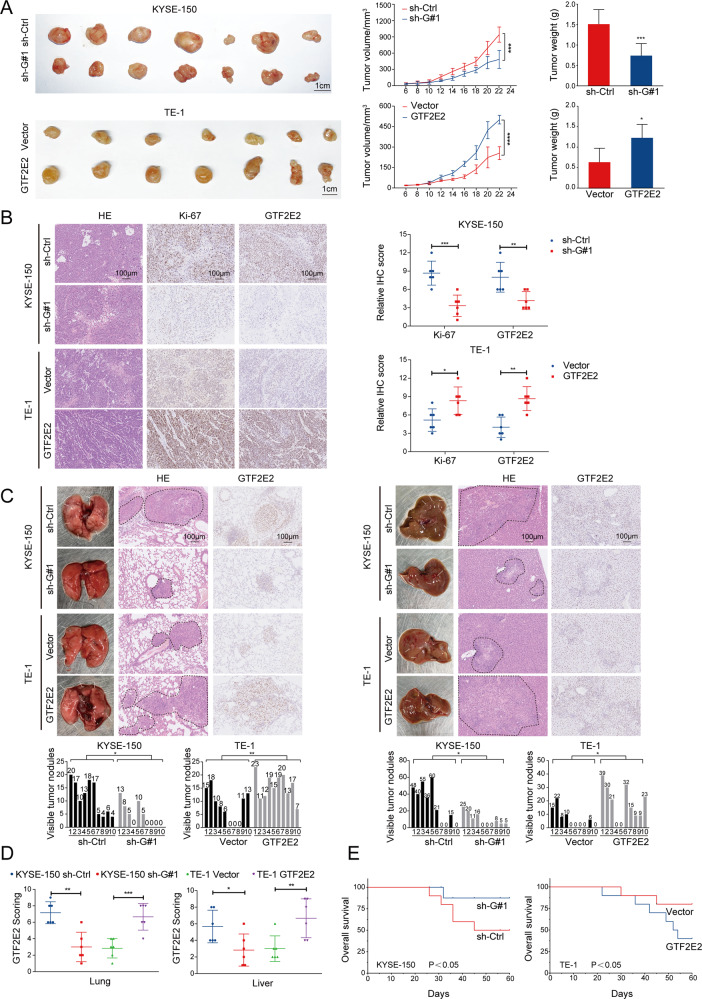
GTF2E2 promotes tumor growth and metastasis of ESCC cells in vivo. **A** The indicated cells were subcutaneously injected into BALB/c nude mice. The volume and weight of subcutaneous tumors in the indicated groups were quantified. **B** Representative images of H&E and IHC staining, and the relative IHC scores (*n* = 6) of Ki67 and GTF2E2 in ESCC tumor xenografts of mice. **C**, **D** ESCC cells were injected into the tail veins of nude mice to establish a metastasis animal model. The lung and liver samples from mice with TE-1 cells overexpressing GTF2E2 had more metastatic nodules than those with control cells, and the opposite results were observed in KYSE-150 cells with GTF2E2 downregulation. Quantification of metastatic nodules during autopsy and H&E staining slides. Representative images of IHC staining and the relative IHC scores (*n* = 6) of GTF2E2 in lung and liver of mice. **E** Survival curves of the indicated metastasis animal groups. *p < 0.05, **p < 0.01, ***p < 0.001, ****p < 0.0001 vs. control. *n* = 10/group.

ESCC cells were injected into nude mice subcutaneously to observe the role of GTF2E2 on tumor growth. The volume and weight of subcutaneous tumors markedly increased after GTF2E2 overexpression in TE-1 cells and decreased after GTF2E2 knockdown in Eca-109 and KYSE-150 cells (Figs. 4A and S2A). The reduction of Ki67 expression was observed in the tumor xenografts derived from Eca-109 and KYSE-150 cells with GTF2E2 depleted, and the opposite results were observed in TE-1 cells with GTF2E2 upregulated (Figs. 4B and S2B), suggesting that GTF2E2 played a vital role in ESCC growth. We then established an ESCC metastasis animal model by injecting cancer cells via the tail vein. More metastatic nodules were observed in post-mortem lungs and livers of mice injected with GTF2E2-overexpressing TE-1 cells compared to those with control cells, and H&E staining was further conducted to verify the formation of metastatic lesions. The opposite results were observed in mice injected with GTF2E2-knockdown Eca-109 and KYSE-150 cells. We investigated the GTF2E2 protein level in the metastatic nodules with IHC staining (Figs. 4C, D and S2C). Furthermore, six mice injected with GTF2E2- overexpressing TE-1 cells died from metastasis within 60 days, while two mice with control cells died within the same period. Conversely, knocking down GTF2E2 in Eca-109 and KYSE-150 cells improved the OS of metastasis model mice (Figs. 4E and S2D). Altogether, the above results demonstrated that GTF2E2 enhances metastasis of ESCC cells and leads to poor survival of mice.

### The mRNA of GTF2E2 is targeted and downregulated by miR-139-5p

**Fig. 5:**
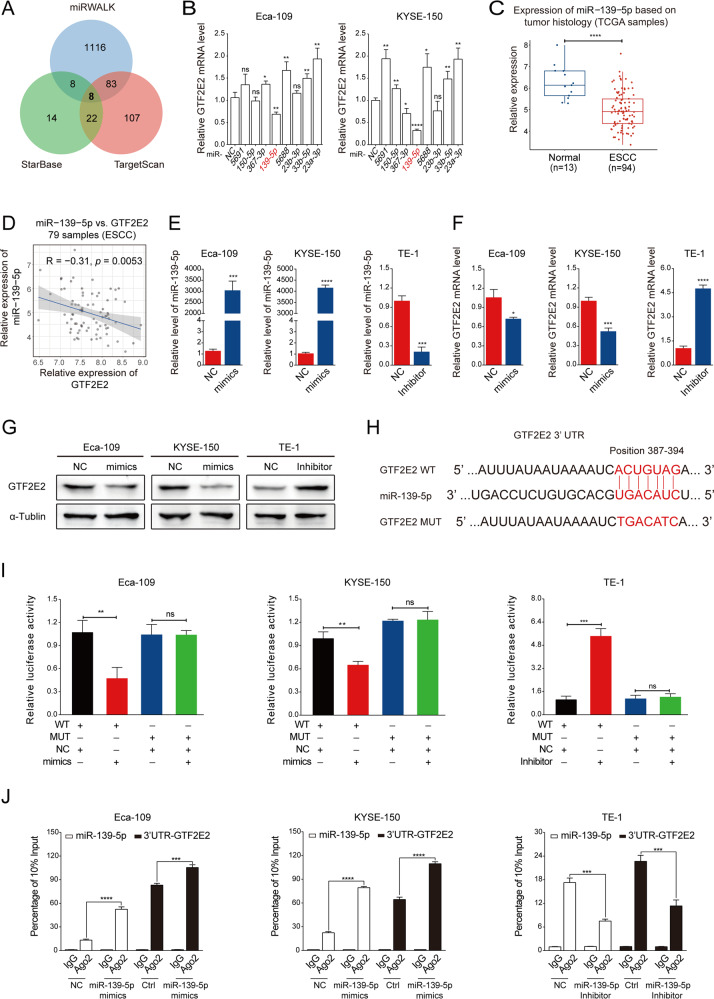
The mRNA of GTF2E2 is targeted by miR-139-5p. **A** Overlap of the predicted miRNAs targeting GTF2E2 mRNA from three different websites. **B** RT-PCR analysis of GTF2E2 mRNA level after transfecting the indicated miRNA mimics for 48 h in Eca-109 and KYSE-150 cells. NC negative control. Data were normalized to GAPDH. **C** Comparison of the miR-139-5p level between ESCC and normal tissues in TCGA database. **D** The correlation analysis of miR-139-5p and GTF2E2 expression in TCGA ESCC samples. **E** RT-PCR analysis of miR-139-5p overexpression (mimics) or downregulation (inhibitor) efficacy in the indicated cells. Data were normalized to U6. **F**, **G** RT-PCR (**F**) and western blot (**G**) analysis of GTF2E2 mRNA and protein level after overexpressing miR-139-5p in Eca-109 and KYSE-150 cells or downregulating miR-139-5p in TE-1 cells. Data were normalized to GAPDH and α-Tublin, respectively. **H** Schematic image of binding site between miR-139-5p and GTF2E2-3′-UTR. **I** The indicated cells were co-transfected with 3′UTR-GTF2E2-WT (or 3′UTR-GTF2E2–MUT) plasmids and miR-139-5p mimic (or miR-139-5p inhibitor). Forty-eight hours after transfection, cells were subjected to dual-luciferase assay. **J** RIP assays of miR-139-5p and 3′UTR-GTF2E2 enrichments by anti-IgG or anti-Ago2 antibodies in miR-139-5p- overexpressing Eca-109 and KYSE-150 cells, and miR-139-5p-downregulated TE-1 cells. Data were normalized to 10% input. *p < 0.05, **p < 0.01, ***p < 0.001, ****p < 0.0001 vs. control. n.s. not significant. *n* = 3.

To find the candidate upstream miRNAs of GTF2E2, we used online bioinformatics websites to predict miRNAs that potentially bind with GTF2E2 mRNA. Eight miRNAs were co-predicted in TargetScan, miRWALK, and StarBase websites (Fig. 5A). We then overexpressed these eight miRNAs, respectively, in Eca-109 and KYSE-150 cells by miRNA mimics. RT-PCR revealed changes in GTF2E2 mRNA level after transfection with these miRNA mimics, and the level of miR-139-5p was negatively correlated with GTF2E2 mRNA in both cell lines (Fig. 5B). Based on the TCGA database, the expression of miR-139-5p was shown to be significantly higher in normal tissues relative to ESCC tumor samples (Fig. 5C). Given miRNA mainly works by binding and decaying targeted mRNA, we analyzed the mRNA expression relevance between GTF2E2 and miR-139-5p in TCGA ESCC samples. The mRNA level of miR-139-5p was found in negative correlation with GTF2E2 mRNA (Fig. 5D). Therefore, we deduced that miR-139-5p is a potential regulator of GTF2E2 in ESCC. Next, we transfected miR-139-5p mimics in Eca-109 and KYSE-150 cells with high endogenous GTF2E2 level and miR-139-5p inhibitor in TE-1 cells with low endogenous GTF2E2 level (Fig. 5E). RT-PCR and western blot assays demonstrated that overexpression of miR-139-5p downregulated and inhibition of miR-139-5p upregulated the GTF2E2 mRNA and protein level (Fig. 5F, G). TargetScan predicted that the position 387–394 of GTF2E2 3′ UTR is a potential binding site of miR-139-5p. We obtained dual-luciferase reporter plasmids 3′UTR-GTF2E2–WT and 3′UTR-GTF2E2-MUT (Fig. 5H). The dual-luciferase reporter assay suggested that miR-139-5p mimics inhibited and miR-139-5p inhibitor enhanced reporter luciferase activity, and the changes were diminished after mutating the binding position (Fig. 5I). The anti-Ago2 RNA immunoprecipitation (RIP) assay suggested that Ago2 enriched miR-139-5p and 3′UTR of GTF2E2. The enrichments increased in miR-139-5p-overexpressing Eca-109 and KYSE-150 cells and decreased in miR-139-5p-downregulated TE-1 cells (Fig. 5J). Therefore, we deduced miR-139-5p is a potential regulator of GTF2E2 in ESCC.

**Fig. 6:**
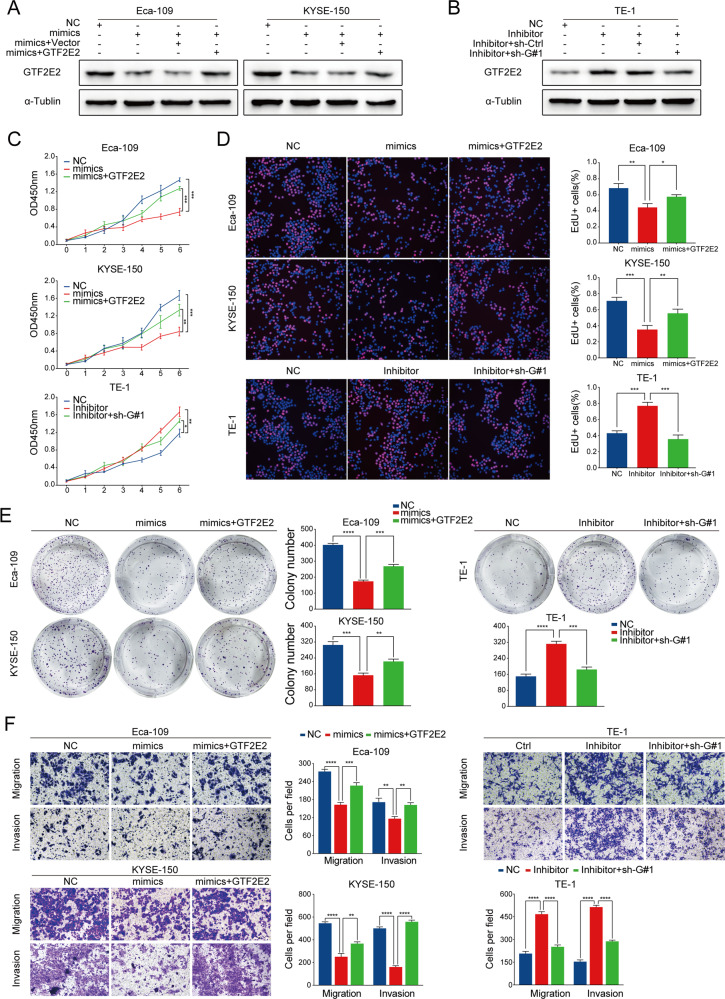
The role of miR-139-5p in ESCC cells was mediated by GTF2E2. **A** GTF2E2 was transfected into miR-139-5p-overexpressing Eca-109 and KYSE-150 cells, and western blot was performed to evaluate GTF2E2 expression. **B** GTF2E2 was stably knocked down in miR-139-5p-downregulated TE-1 cells, and western blot was performed to analyze the GTF2E2 level. α-Tublin was used as a loading control. **C**–**E** CCK8, EdU, and colony formation assays were conducted in the indicated cells. Quantification of colony numbers and percentages of Edu+ cells. **F** Representative images of transwell assay in the indicated cells and quantification of migrated and invaded cells. *p < 0.05, **p < 0.01, ***p < 0.001, ****p < 0.0001 vs. control. *n* = 3.

We further overexpressed GTF2E2 in Eca-109 and KYSE-150 cells with upregulated miR-139-5p, and stably knocked down GTF2E2 in TE-1 cells with downregulated miR-139-5p (Fig. 6A, B). CCK8, EdU, and colony formation assays revealed that overexpression of GTF2E2 reversed the inhibited cell proliferation in miR-139-5p-upregulated cells. Transwell assay showed that the impaired migration and invasion abilities in miR-139-5p-upregulated cells were recovered by overexpression of GTF2E2. In contrast, the enhanced proliferation, migration, and invasion abilities in miR-139-5p-downregulated cells were impeded by knockdown of GTF2E2 (Fig. 6C–F).

### Identification of whole-genome DNA-binding sites and transcription targets for GTF2E2 by RNA-seq and ChIP-seq

**Fig. 7:**
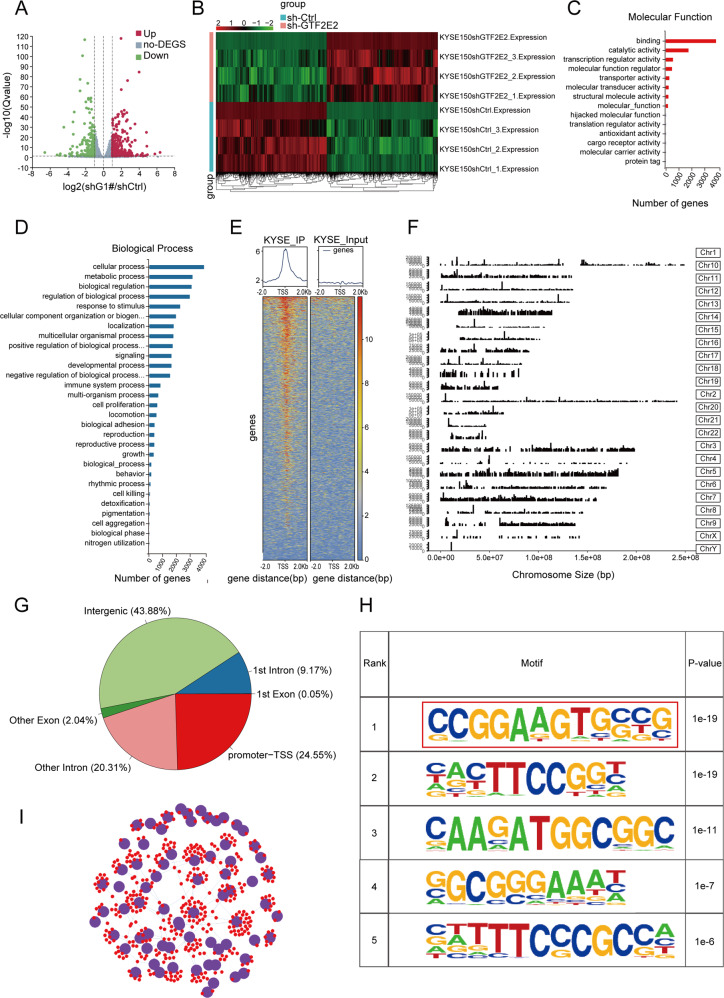
Identification of whole-genome DNA-binding sites and transcription targets for GTF2E2 by RNA-seq and ChIP-seq. **A** Differential mRNA expression profiles between GTF2E2-ctrl and GTF2E2 downregulation. Red = genes with higher expression, green = genes with lower expression, and gray = genes with no statistical difference. **B** Heat map displays the differentially expressed genes between GTF2E2-ctrl and GTF2E2 knockdown. **C**, **D** The important GO terms for molecular functions and biological processes by GO analysis. **E** The reads distributed on two sides of the transcription start site (TSS). **F** ChIP peaks over chromosomes by ChIP-seq technology employing the primary antibody against GTF2E2. **G** Pie diagram shows the ratios of GTF2E2-binding sites located relative to a transcription unit including intergenic, 1st intron, 1st exon, promoter-TTS, other intron and other exon. **H** Five predicted GTF2E2-binding motifs with the most significant differences among peaks. **I** The network diagram between TFs and genes interaction based on the results of ChIP-seq. Purple represents the transcription factor or transcription factor family, and red represents the target gene that TF may interact with through motifs. *n* = 3.

To assess the impact of GTF2E2 on gene expression, we performed transcriptome sequencing (RNA-Seq) analysis in both control and GTF2E2-downregulated cells and identified the differentially expressed genes. Combined with the *p* value and fold change (FC), we found that GTF2E2 knockdown resulted in upregulation of 2261 genes and downregulation of 2263 genes (Fig. 7A). A visualization of the differential expression pattern of the genes is displayed using a hierarchical clustering heat map (Fig. 7B). Gene Ontology (GO) analysis was applied to examine biological functions, and the critical GO terms for molecular functions and biological processes are provided in Fig. 7C–D. The terms were related to metabolism, immune system process and tumorigenesis, such as cell proliferation and growth. The same gene set was also explored using Kyoto Encyclopedia of Genes and Genomes (KEGG) analysis. The significant pathways are presented in Supplementary Fig. S3A, with pathways relating to cancer and apoptosis well represented in the deregulated genes. The phosphorylation level of AKT, ERK, and mTOR were evaluated by western blot in the indicated ESCC cells (Fig. S3B).

We then studied the genome-wide target sites of GTF2E2 in ESCC cells using the ChIP-seq approach and identified 27,149 peaks corresponding to 7503 RefSeq genes (Fig. 7E). The peaks over chromosomes suggested different peak values. The abscissa indicates the chromosome size, the right ordinate shows the chromosome number, and the left ordinate represents each chromosome peak value (Fig. 7F). GTF2E2 was preferentially located near the promoter-transcription start sites (TSSs) of genes (Fig. 7G). GO analysis of the peak-related genes suggested that the GTF2E2 target genes were involved in various biological processes, such as translation initiation, metabolism, and kinase activity regulation (Fig. S3C). KEGG analysis showed that the GTF2E2 peak-related genes were markedly enriched in cell cycle and PI3K–Akt pathways (Fig. S3D). The motifs in common between the peaks were detected, and the five motifs with the most significant differences are shown (Fig. 7H). The motifs could bind to many TFs, and an interaction network diagram is shown according to the corresponding relationship between TFs and genes (Fig. 7I).

### FUS is identified as a target of GTF2E2 and mediates the GTF2E2-induced progression in ESCC cells

**Fig. 8:**
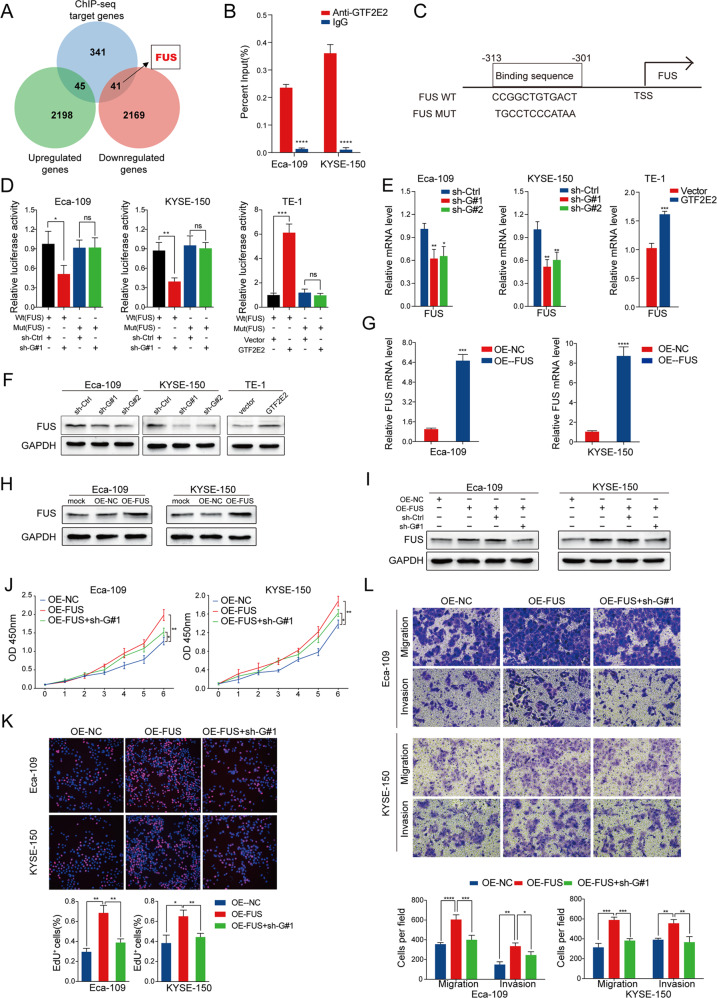
FUS is identified as a target of GTF2E2 and mediates the GTF2E2-induced progression in ESCC cells. **A** The Venn diagram shows the number of overlapping genes between differentially expressed gene sets by GTF2E2 knockdown through RNA-seq and the target gene set identified by ChIP-seq. **B** ChIP-PCR was used to detect the binding of GTF2E2 to the FUS promoter in Eca-109 and KYSE-150 cells using GTF2E2 primary antibody, and IgG was applied as a negative control. **C** Putative wild and mutant binding sites between GTF2E2 and the FUS promoter. **D** Dual-luciferase assay was performed using firefly luciferase reporter vectors and Renilla luciferase was used as an internal control. **E**, **F** Validation of FUS expression changes using RT-PCR (**E**) and western blot (**F**) analysis after upregulated or downregulated GTF2E2 expression in ESCC cell lines. GAPDH was used as a loading control. **G**, **H** RT-PCR and western blot analysis of FUS overexpression efficiency in the indicated cells. GAPDH was used as a loading control. **I** pcDNA3.1-FUS plasmids were transfected into GTF2E2-downregulated ESCC cells, and FUS expression was confirmed by western blot. **J**, **K** CCK8 and EdU assays were performed to determine the proliferation impact of ESCCs treated with GTF2E2 and FUS or negative control. Quantification of percentages of Edu+ cells. **L** Transwell analysis showed the migration and metastasis capacity of ESCC cells co-transfected with GTF2E2 and FUS or negative control. Quantification of migrated and invaded cells. *p < 0.05, **p < 0.01, ***p < 0.001, ****p < 0.0001 vs. control. *n* = 3.

Next, we investigated the overlapping gene sets between the differentially expressed genes after GTF2E2 knockdown and ChIP-seq. It was found that 45 upregulated genes were included in the set of GTF2E2 target genes, while 41 downregulated genes were included in the set of GTF2E2 target genes (Fig. 8A). Interestingly, we found that FUS, an oncogene factor, was among the group of 41 downregulated target genes. Based on the TCGA database, ESCC tissues expressed FUS at significantly higher level relative to adjacent counterparts (Fig. S4A), demonstrating the potential oncogenic activity of FUS in ESCC. Then, we analyzed the mRNA expression relevance between GTF2E2 and FUS in TCGA ESCC samples. The mRNA level of FUS was found in positive correlation with GTF2E2 mRNA (Fig. S4B). According to the ChIP-seq results, we designed the primers of FUS promoter and performed ChIP followed by RT-PCR to confirm that GTF2E2 bound directly to the promoter region of FUS in Eca-109 and KYSE-150 cells, and IgG was applied as a negative control (Fig. 8B). With the help of bioinformatics tools, we compared all predicted motifs with the promoter-binding region of FUS, and it was found that the first motif labeled in Fig. 7H might be a potential binding site. The putative wild and mutant binding sites between GTF2E2 and FUS promoter are shown in Fig. 8C. Dual-luciferase reporter assay suggested that GTF2E2 downregulation inhibited and GTF2E2 upregulation enhanced reporter luciferase activity, and the changes were diminished after mutating the FUS promoter-binding position (Fig. 8D).

Furthermore, we evaluated whether GTF2E2 promotes ESCC progression through FUS. RT-PCR and western blot results showed that GTF2E2 downregulation inhibited and GTF2E2 upregulation enhanced FUS expression level (Fig. 8E, F). Then, we successfully overexpressed FUS using pcDNA3.1-FUS plasmids (Fig. 8G, H) and stably knocked down FUS by lentivirus transduction with three independent shRNAs (Fig. S4C, D) in Eca-109 and KYSE-150 cells confirmed by RT-PCR and western blot. We further upregulated FUS in GTF2E2-downregulated cells (Fig. 8I) and transfected a FUS-downregulating lentivirus in GTF2E2-upregulated ESCC cells (Fig. S4E) confirmed by western blot. FUS overexpression restored the changes in CCK8, EdU, and transwell assay induced by GTF2E2 downregulation (Fig. 8J–L). In contrast, the enhanced proliferation, migration, and invasion abilities in GTF2E2-upregulated cells were impaired by knockdown of FUS (Fig. S4F–H). In western blot analysis, FUS downregulation or upregulation restored the changes in EMT protein markers induced by GTF2E2 overexpression or knockdown (Fig. S4I, J). The reduction of FUS expression was observed in the tumor xenografts derived from Eca-109 and KYSE-150 cells with GTF2E2 downregulation, and the opposite trend was found in the tumor xenografts derived from TE-1 cells with GTF2E2 upregulation (Fig. S5A). These suggesting that FUS played a vital role in GTF2E2-mediated ESCC growth. Then, we explored FUS protein level in the metastatic nodules with immunohistochemistry (IHC), and found that FUS was significantly reduced in the lung and liver metastatic nodules from ESCC cells with downregulated GTF2E2. The opposite trend was observed in mice injected with GTF2E2 upregulated TE-1 cells (Fig. S5B, C). Moreover, in Eca-109 and KYSE-150 cells, western blot analysis showed that FUS downregulation or upregulation restored the changes in the phosphorylation level of AKT, ERK, and mTOR induced by GTF2E2 overexpression or knockdown (Fig. S6A, B). These results confirmed that GTF2E2 promotes ESCC progression through FUS.

## Discussion

The dysregulated expression of oncogenes or tumor suppressor genes plays a crucial role in ESCC tumorigenesis and progression. Currently, although surgical resection is still the mainstay treatment for potentially curable esophageal cancer, most tumors recurred within 1 year after surgery, which highlights the significance of exploring promising biomarkers for early detection of recurrence [31–33]. Our study presents a novel insight into GTF2E2’s potential function as a postoperative recurrence marker in the ESCC. Through bioinformatical analysis and IHC staining in tumor tissue microarray, we found that GTF2E2 was overexpressed in tumor tissues of ESCC compared with that in adjacent non-tumor tissues. GTF2E2 overexpression is associated with advanced N stage and clinical stage in ESCC, and could predict worse prognosis and shorter survival in ESCC patients. Furthermore, a higher GTF2E2 level was observed in the early postoperative recurrent ESCC samples compared to the late recurrent ESCC samples. Both in vitro and in vivo results suggested that knockdown of GTF2E2 significantly inhibited cell proliferation and metastasis of ESCC cells, and these effects were effectively promoted by GTF2E2 overexpression. These phenomena might be associated with the deregulation of miR-139-5p and consequently enhanced expression of FUS. Besides, we suggested that GTF2E2 promotes ESCC cells progression via activation of the AKT/ERK/mTOR signaling pathway. Together, this study showed that GTF2E2 might act as an oncogene and negative prognostic factor in ESCC, thus potentially serving as a promising biomarker for early detection of recurrence in ESCC patients after surgery.

As a significant component for RNA transcription initiation, GTF2E2 is involved in various biological processes, and its alterations are related to diverse pathological conditions, such as trichothiodystrophy [34, 35] and viral replication [36]. Previous studies have reported that GTF2E2 is expressed aberrantly in several cancers and correlates with poor prognosis. For example, Yang et al. [15] reported that GTF2E2 was involved in glioblastoma progression by increasing the level of the cell division cycle 20 (CDC20) in their bioinformatical analysis. Bi et al. [17] showed that GTF2E2 facilitated lung adenocarcinoma progression by interacting with RPS4X, which correlated with poor clinical outcomes. However, the biological functions of GTF2E2 in ESCC tumor progression largely remain unclear. In the present study, we explored the oncogenic role of GTF2E2 in ESCC tumorigenesis and metastasis by gain- and loss-of function research in vitro and in vivo. Overexpression of GTF2E2 facilitates migration and invasion of ESCC cells, and we conducted western blot and immunofluorescence assays to examine the EMT markers and found that overexpressed GTF2E2 can significantly affect the levels of EMT markers in vitro. Notably, GTF2E2-overexpressed cells promote lung and liver metastasis of ESCC cells in nude mice, as proven by H&E staining. This suggested that GTF2E2 is involved in the process of mesenchymal–epithelial transition (MET). MET is the reversion of EMT and is apparent during development, induced pluripotent stem cell reprogramming, and tumor metastasis. In the multistep process of tumor invasion and metastasis, EMT is believed to dominate the early stage of the metastatic cascade, whereas MET is the dominant terminal stage [37]. We need to explore whether GTF2E2 is involved in the transformation of EMT and MET, using lineage tracing of cells from their origin to their ultimate destination in further study. Furthermore, future research will be required to explore the role of GTF2E2 in clinical practice.

In our research, bioinformatics analysis predicted that the mRNA of GTF2E2 could be targeted by serials of miRNAs. Among these miRNAs, we identified miR-139-5p as an upstream regulator of GTF2E2 by analyzing the expression relevance between miRNAs and GTF2E2 in ESCC cells. GTF2E2 was downregulated, whereas miR-139-5p was upregulated, which were negatively correlated with each other. We further found that miR-139-5p downregulated GTF2E2 expression through binding with Ago2, the core protein of RISC complexes. Our results suggested that upregulated GTF2E2 markedly restored the proliferation, migration, and invasion of ESCC cells inhibited by miR-139-5p overexpression in the in vitro experiment, indicating that the effects of miR-139-5p in ESCC cells were mediated by GTF2E2. miR-139-5p is a well-known tumor suppressor in several types of cancer cells [38–40]. The expression of miR-139-5p is reduced in esophageal cancer and it acts as a tumor suppressor, which is in line with our study [22–25].

RNA-seq and ChIP-seq data predicted that FUS is the direct target of GTF2E2, which is a well-known oncogene [28–30]. In the current study, we hypothesized that GTF2E2 might regulate the transcription of FUS in ESCC. Accordingly, the effect of GTF2E2 on the FUS transcription was analyzed. The putative binding sites between GTF2E2 and FUS promoter were analyzed using bioinformatics tools, and mechanism experiments showed the binding of GTF2E2 to FUS promoter. Combining the above with the positive regulation of GTF2E2 on FUS expression, we confirmed that GTF2E2 transcriptionally activates FUS. Finally, rescue assays demonstrated the reversal effect of upregulated or downregulated FUS on the cell proliferation, migration, and invasion mediated by the GTF2E2 knockdown or overexpression in ESCC.

Previous studies have fully confirmed the signaling pathways involved in tumor cell progression, including mTOR, PI3K-AKT, and MAPK/ERK1/2 [41–43]. To determine the downstream mechanisms of GTF2E2 in ESCC, the potential involvement of the signaling pathway was assessed. Combined with the results of KEGG analysis, we found that GTF2E2 could activate AKT/ERK/mTOR pathway to promote the aggressive biological behaviors of ESCC cells.

In this study, we illustrate for the first time that GTF2E2 plays an oncogenic role in ESCC progression with clinical analysis and both in vivo and in vitro experiments.

Data gathered in our study suggested GTF2E2 promotes ESCC progression via the miR-139-5p/GTF2E2/FUS axis. Furthermore, the activation of EMT, MET, and the AKT/ERK/mTOR signaling pathway caused by GTF2E2 manipulation were recovered by rescuing FUS expression in ESCC cells. The miR-139-5p/GTF2E2/FUS axis may affect ESCC cell proliferation and invasion via the acceleration of EMT, MET, and the phosphorylation of AKT/ERK/mTOR signaling pathway components. Altogether, our study provides the evidence that GTF2E2 is a potential biomarker for postoperative recurrence and a therapeutic target in patients with ESCC.

## Materials and methods

### Patients and ESCC samples

In this study, tissue microarray of ESCC samples and adjacent non-tumor tissues were obtained from Shanghai Outdo Biotech Co., Ltd. Patients who experienced recurrence within 1 year after surgery were defined as the early recurrence group and the remaining patients with recurrence were defined as the late recurrence group [31–33]. Thirty-five archived formalin-fixed paraffin-embedded (FFPE) early postoperative recurrence ESCC samples and 106 FFPE late postoperative recurrence ESCC samples were obtained from the Department of Thoracic Surgery, Tongji Hospital, Tongji Medical College, Huazhong University of Science and Technology, Wuhan, China between 2015 and 2017 in accordance with the principles of the Declaration of Helsinki. The Ethics Committee of Tongji Hospital approved the study, and informed consent was obtained from all patients.

### Cell culture and transfection

The human ESCC cell lines KYSE-150 and TE-1 were purchased from the cell bank of the Chinese Academy of Sciences (Shanghai, China). Eca-109 was purchased from the National Infrastructure of Cell Line Resource (Beijing, China). KYSE-410, KYSE-30, KYSE-450, and normal human esophageal epithelial cell line HEEC were maintained at the Laboratory of Oncology in Tongji Hospital. All cell lines were authenticated recently by short tandem repeats (STRs) profiling and confirmed to be mycoplasma negative. They were maintained in RPMI 1640 (Hyclone, United States), supplemented with 10% fetal bovine serum (FBS, Gibco, United States) and 1% penicillin/streptomycin (Hyclone, United States).

The miRNA mimics and inhibitors, and normal (scramble) control (NC) oligonucleotides were obtained from Ribobio (Guangzhou, China). A FUS expression construct was created by subcloning the PCR-amplified full-length human FUS cDNA into the pcDNA3.1 vector (Ribobio, Guangzhou, China). miRNA or plasmids transfection was performed using Lipofectamine™ 3000 transfection reagent (Invitrogen, USA) according to the manufacturer’s instructions. The short hairpin RNAs (shRNAs) specific to GTF2E2 and FUS (sh-GTF2E2#1/2/3 and sh-FUS#1/2/3) were synthesized and cloned into lentiviral vectors by Genechem Co., Ltd. (Shanghai, China). Lentivirus containing full-length GTF2E2 cDNA was also synthesized and transfected into ESCC cells to construct GTF2E2-overexpressing cell lines. Cell transfection was performed according to the manufacturer’s protocol. Target sequences are provided in Table S1.

### Dual-luciferase assay

To confirm the direct regulating relationship between miR-139-5p and GTF2E2, the 3′-untranslated region (3′UTR) of GTF2E2 or 3′UTR-GTF2E2-mutant was cloned into the pmiR-RB-Report™ vector (Ribobio, Guangzhou, China). About 1 × 10^5^ cells/well were seeded into 24-well plates. After 24 h, the recombinant plasmid 3′UTR-GTF2E2-WT or 3′UTR-GTF2E2-mutant was co-transfected into cells with miR-139-5p mimic, miR-139-5p inhibitor, or their respective negative control (NC) using Lipofectamine 3000 (Invitrogen). The transfected cells were incubated for 48 h and total protein from cells was extracted by Passive Lysis Buffer (Promega), and the luciferase activity was examined using Dual-Luciferase® Reporter Assay System (Promega) on GloMax 20/20. Firefly luciferase activity was normalized against Renilla luciferase values, and the ratio of firefly/Renilla luciferase activity was presented.

To perform FUS promoter analysis, cells were seeded into 24-well plates. FUS promoter-binding sequence (WT or MUT) was cloned into pPRO-RB-Report vector (Ribobio, Guangzhou, China). Cells were co-transfected with the plasmids containing FUS promoter-binding sequence (WT or MUT) and GTF2E2 upregulation, downregulation, or negative control using Lipofectamine 3000 (Invitrogen). After 48 h, the relative luciferase activity was measured with the Dual-Luciferase Reporter Assay System (Promega).

### Anti-Ago2 RNA-binding protein immunoprecipitation (RIP) assay

The antibody against Ago2 was purchased from Abcam (ab186733). Magna RIP™ RNA-Binding Protein Immunoprecipitation Kit (Millipore, Darmstadt, Germany) was applied to enrich Ago2-binding RNA as demonstrated in the manufacturer’s instructions. The enriched RNA was analyzed by RT-PCR. 2^−ΔCT^ was calculated and normalized to the 2^−ΔCT^ of 10% input. The primer sequence of GTF2E2-3′ UTR is as follows: forward: TGCATTTACGGGAAAGGGCT; reverse: AGACCCTTCCTTGTCCCACA.

### ChIP, ChIP-seq, ChIP-PCR

*ChIP*: chromatin immunoprecipitation (ChIP) assay was conducted using the SimpleChIP Enzymatic Chromatin IP Kit (#9003, CST) according to the manufacturer’s instructions. In short, cells were crosslinked with 37% formaldehyde, and fragmented chromatin was treated with nuclease and subjected to sonication. Consequently, the supernatants were collected and incubated overnight with anti-GTF2E2 antibody (ab228581, Abcam), anti-histone H3 antibody (a technical positive control; #4620, CST), and normal rabbit IgG antibody (a negative control; #2729, CST) combined with ChIP-Grade Protein G magnetic beads. The beads were washed, and the chromatin complexes were harvested, purified, and reverse-crosslinked, followed by DNA purification, and ChIP-seq and ChIP-PCR detection.

*ChIP-seq*: The immunoprecipitated DNA was analyzed through deep sequencing (ChIP-seq) by SEQHEALTH Biotech Company (Wuhan, China). ChIP libraries were prepared using ChIP DNA according to the BGISEQ-500ChIP Seq library preparation protocol. After filtering the raw sequencing data (raw data), high-quality sequencing data (clean data) were compared to the human genome (hg19) to obtain the ChIP-seq results. Next, the results were compared to the whole-genome de novo peak calling to explore the protein’s interacting preference in the genome and to perform motif analysis of the binding site.

*ChIP-PCR*: The immunoprecipitated DNA was quantified by real-time PCR with primers for GTF2E2-binding sites in the FUS promoter (forward: GTGGAGATAGATCGTGGGCTAGT; reverse: CAGGCAGAAGGGAAACAAGTTA). Fold enrichment was analyzed based on the threshold cycle (CT) value of the IgG control using the comparative CT method.

### Animal experiments

Experiments have been conducted according to the ethical standards, the Declaration of Helsinki, and national and international guidelines. All animal research was conducted according to the ARRIVE (Animal Research: Reporting of In Vivo Experiments) guidelines and the AVMA (the American Veterinary Medical Association) guidelines on euthanasia and was approved by the Animal Ethics Committee of Tongji Hospital, Tongji Medical College, Huazhong University of Science and Technology.

*Tumor formation assay*: female BALB/c nude mice of 4–5 weeks old (Charles River, Beijing, China) were maintained at SPF conditions, and given a subcutaneous injection of 5 × 10^6^ cells (groups: sh-Ctrl, sh-G1#; OE-Ctrl, OE-GTF2E2) into the right flank, respectively (10 mice/group). The mice were randomly divided into groups before injection. Then the subcutaneous xenografts were measured every 2 days. Tumor volume was measured using calipers and calculated according to the formula: *V* = length × width^2^/2. After 21 days, the mice were euthanized and the formed tumors were isolated and weighed for the following analyses.

*Metastasis assay*: female BALB/c nude mice of 4–5 weeks old were injected with 100 μL of cell suspension containing 1 × 10^7^ cells via the tail vein (10 mice/group). After 8 weeks of cells injection, the mice were sacrificed, and the lungs and livers were removed to calculate metastatic nodules.

### IHC analysis

The paraffin-embedded tissue samples were sliced into 5-μm‐thick sections, and all slides were stained with GTF2E2 (ab187143, Abcam), Ki67 (ab16667, Abcam), or FUS (ab124923, Abcam) according to the standard procedures. Then GTF2E2 expression was scored according to the staining scope and intensity. Specifically, the staining scope was: 1 (0–25%); 2 (25–50%); 3 (50–75%); and 4 (75–100%), and the staining intensity was 0 (negative); 1 (weakly positive); 2 (moderately positive); and 3 (strongly positive). The overall score was defined by multiplying the staining scope by the staining intensity score [44]. IHC staining scores were independently analyzed by three pathologists without prior knowledge of patient characteristics.

### Online bioinformatics analysis

The online bioinformatics websites, TargetScan [45], miRWALK [46], and StarBase 2.0 [47] were used to predict miRNAs targeting GTF2E2 mRNA.

### Statistical analyses

Data analysis was performed using SPSS Statistics 25.0. The paired *t*-test was applied to detect the differential expression of GTF2E2 in cancer tissues compared with adjacent non-malignant tissues, and in early postoperative recurrence samples compared with late postoperative recurrence samples. The relationship between GTF2E2 and clinicopathological characteristics was analyzed using the chi-square test. Survival curves were evaluated using Kaplan–Meier and log-rank tests. The effects of variables on survival were analyzed by the univariate and multivariate Cox proportional hazards models. Two-group or multiple-group comparison was calculated with Student’s *t*-test or one-way ANOVA, followed by the Dunnett’s post hoc test using GraphPad Prism 5 software (GraphPad Software, USA). All in vitro experiments were conducted three times. All values were shown as mean ± SD. Differences were defined as statistically significant at *p* < 0.05 (*p < 0.05; **p < 0.01; ***p < 0.001).

## Supplementary information

supplementary

Table S2
