# Supplementary material for: GTF2E2 is a novel biomarker for recurrence after surgery and promotes progression of esophageal squamous cell carcinoma via miR-139-5p/GTF2E2/FUS axis
Source: Oncogene. 2021 Dec 2;41(6):782–96. doi: 10.1038/s41388-021-02122-8 (PMC8816730; doi:10.1038/s41388-021-02122-8)
Supplement: Supplementary file 1 — supplementary [file 41388_2021_2122_MOESM1_ESM.docx]

# Additional information

**RNA Extraction and Quantitative Real‑time Polymerase Chain Reaction (RT‑PCR)**

Total RNA was harvested from cells using RNAiso Plus (Takara, Japan), and cDNA was synthesized by 1 μg RNA using PrimeScript RT Master Mix (RR036A; Takara, Japan) according to the manufacturer's instructions. RT-PCR was performed as described previously (1). The primer sequences are as follows: GTF2E2 (forward: TAGAACATGGAGGATCGTCAGG; reverse: CGCTGATGCCGTGTCTTCA), FUS (forward: ATGGCCTCAAACGATTATACCCA; reverse: GTAACTCTGCTGTCCGTAGGG), and GAPDH (forward: GGAGCGAGATCCCTCCAAAAT; reverse: GGCTGTTGTCATACTTCTCATGG).

# Western Blot Analysis

Western blot was performed in accordance with standard procedures as described previously (1). In our study, the following antibodies were used: the antibodies against E-Cadherin (24E10) (#3195), N-Cadherin (D4R1H) (#13116), Vimentin (D21H3) (#5741), β-Catenin (D10A8) (#8480)

and Snail (C15D3) (#3879) obtained from Cell Signaling Technology (Beverly, MA); the antibodies against GAPDH (#60004-1-Ig) and alpha Tublin (#11224-1-AP) obtained from Proteintech (Wuhan, China); and the antibodies against GTF2E2 (ab187143) and FUS (ab124923) purchased from Abcam (Cambridge, MA).

# Immunofluorescence

Cultured cells were fixed in 4% paraformaldehyde, permeabilized with 0.1% Triton X-100 (Servicebio, Wuhan, China), blocked with 0.5% BSA (Sigma, United States), and incubated with

antibodies against E-cadherin, Vimentin, and β-catenin (Cell Signaling Technology) and the secondary antibody (Proteintech, Wuhan, China), followed by staining with 4′, 6-diamidino-2- phenylindole (DAPI, Boster, Wuhan, China). Finally, cell images were captured by a fluorescence microscope (Leica, Germany), and quantitated using ImageJ software.

# Cell Counting Kit 8 Assay and EdU Incorporation Assay

Cell Counting Kit-8 assay (CCK-8, MedChemExpress, United States) was performed according to the manufacturer's instructions. In short, 1,000 indicated cells were seeded into 96-well plates. The culture medium was changed to 100 μl 10% CCK8 solution at the indicated time and incubated for 2 h at 37°C. Optical density (O.D.) was measured at 450 nm by the microplate reader (BioTek, United States).

ESCC cells (4,000 cells/well) were seeded into 96-well plates and cultured overnight for EdU incorporation assay using Cell-Light™ EdU Apollo567 In Vitro Kit (Ribobio, Guangzhou, China) according to the manufacturer's protocol. In short, 100 μl of 50 μM EdU solution was added to cells for incubation for 2 h. The cells were washed with PBS, fixed with 4% paraformaldehyde and incubated with 0.5% TritonX-100. Then, the cells were stained with 100 μl of 1X Apollo solution for 30 min, and the nuclei were stained with 1X Hoechst33342 solution. Representative images were captured by a fluorescence microscope (Leica, Germany), and positive cells were counted by Image Pro. Plus version 6.0.

# Colony Formation Assay

Cells were seeded in triplicate into 6-well plates (500 cells/well) and incubated for 14 days, fixed with paraformaldehyde for 15 min and stained with 0.1% crystal violet (Servicebio, Wuhan, China) for 20 min. Colonies including outnumbered 50 cells were counted under a microscope.

# Wound Healing Assay

Cells were seeded in triplicate into 24-well plates. When the cells reached a confluence > 90%, the sterile micropipette tips were applied to generate scratch wounds, and the 10% FBS medium was replaced with serum-free RPMI 1640. The scratch width was photographed under a microscope, and the results were presented as percent scratch closure.

# Transwell Assay

Cell invasive capacity was examined with Matrigel-coated Transwell chamber (Corning, United States) in accordance with the supplier's instructions. Cell migration ability was detected using Transwell chamber without Matrigel. Cells were seeded at a density of 6,000 cells into the upper chamber with FBS-free RPMI 1640 and the complete medium was placed into the lower chamber. Cells migrating or invading through the membrane were fixed in 4% paraformaldehyde and stained with crystal violet. Five fields from each well were randomly captured and counted using a microscope (magnification, ×200).

# Cell Apoptosis Assay

Cells were collected and washed with ice-cold PBS. Then, the cells were stained with Annexin V-fluorescein isothiocyanate and PI as suggested in the manufacturer's protocol (BD Biosciences, United States), and analyzed by flow cytometry. The apoptotic cells were determined using FlowJo software.

# RNA-seq

Total RNA from the KYSE-150 cells with GTF2E2 knockdown and control cells were isolated and quantified. The concentration of each RNA sample was measured with a NanoDrop 2000 (Thermo Scientific, USA). The quality was assessed by an Agilent2200 (Agilent, USA). The sequencing library of each RNA sample was prepared using the Ion Proton Total RNA-Seq Kit v2 according to the protocol provided by the manufacturer (Life Technologies, USA). The RNA sequencing was analyzed by the BGI Biotech company (Shenzhen, China).

**References:**

1.Zhang Y, Zhang Y, Li Y, Zhang L, Yu S. Preclinical Investigation of Alpinetin in the Treatment of Cancer-Induced Cachexia via Activating PPARgamma. FRONT PHARMACOL. [Journal Article]. 2021 2021-01-20;12:68749

# Table S1. The target sequences of all the shRNAs used in this study.

| shRNA | Sequence |
| --- | --- |
| sh-GTF2E2#1 | 5’-CCTACTTAGGCTCTTAGAT-3’ |
| sh-GTF2E2#2 | 5’-TTTAGTCAACAATCCCAAA-3’ |
| sh-GTF2E2#3 | 5’-CAGCGAGGATTAGGAGGAATT-3’ |
| sh-FUS#1 | 5’-GCTGATTACTTCAAGCAGATT-3’ |
| sh-FUS#2 | 5’-CGTGGTGGCTTCAATAAATTT-3’ |
| sh-FUS#3 | 5’-CCAGAGCAGCTATTCTTCTTA-3’ |

**
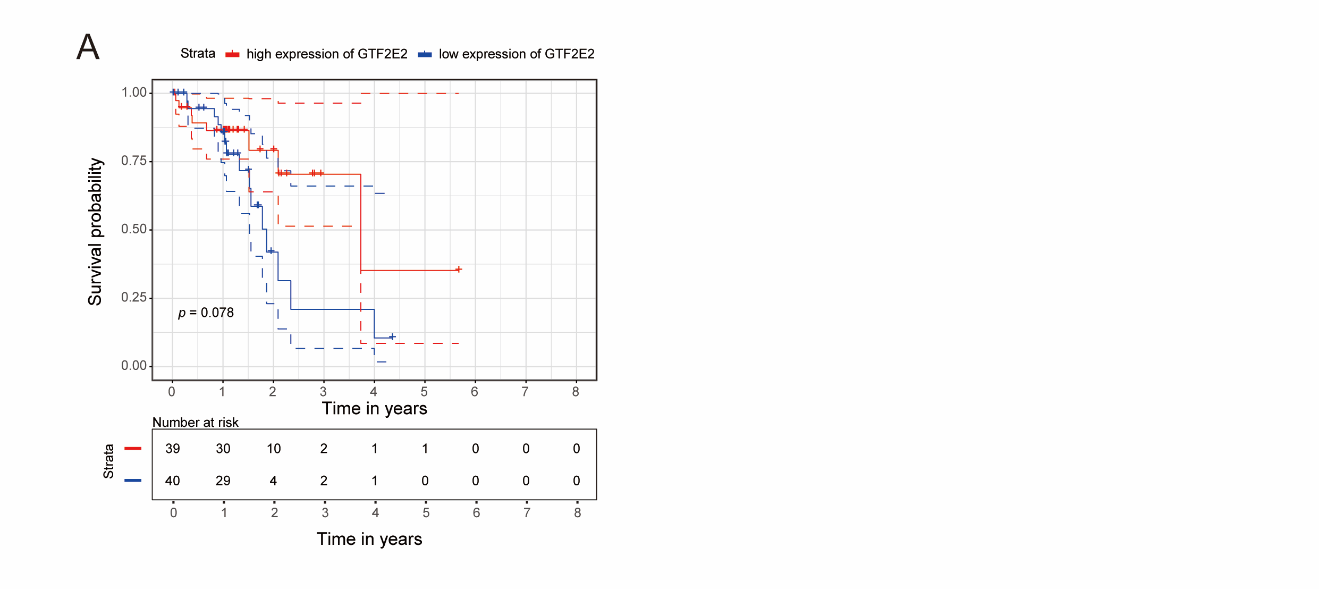
Figure S1. Survival analysis of GTF2E2 expression in ESCC patients from TCGA database.** A. The overall survival was compared between GTF2E2 high (n =39) and low expression (n = 40) in ESCC patients from TCGA cohort.

#
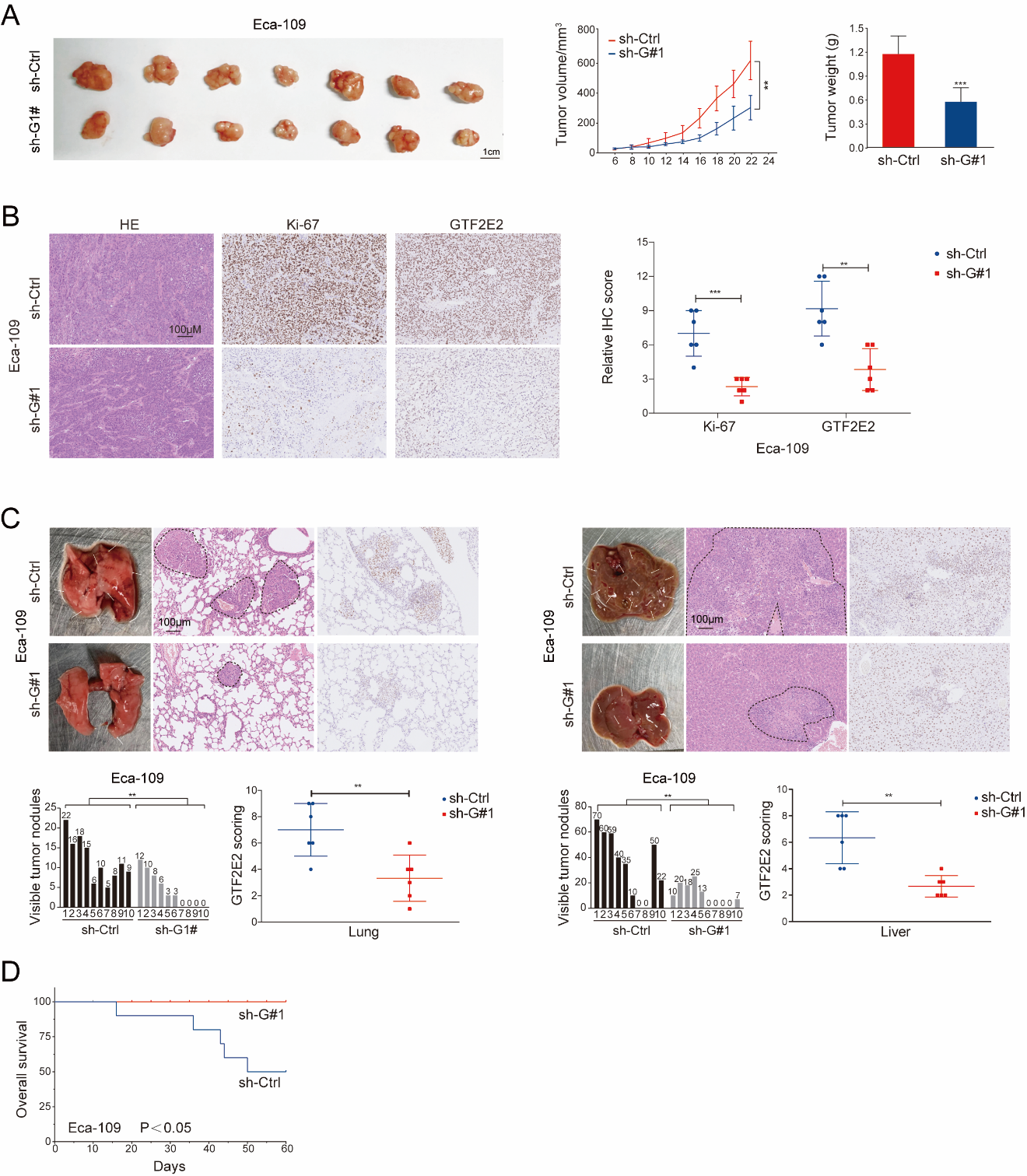
Figure S2. GTF2E2 promotes tumor growth and metastasis of ESCC cells *in vivo*. A. The indicated cells were subcutaneously injected into BALB/c nude mice. The volume and weight of subcutaneous tumors in the indicated groups were quantified. B. Representative images of H&E and IHC staining, and the relative IHC scores (n = 6) of Ki67 and GTF2E2 in ESCC tumor xenografts of mice. C. Eca-109 shCtrl or Eca-109 shG#1 cells were injected into the tail veins of nude mice to establish a metastasis animal model. The lung and liver samples from mice with Eca- 109 cells downregulated GTF2E2 had less metastatic nodules than those with control cells. Quantification of metastatic nodules during autopsy and H&E staining slides. Representative images of IHC staining and the relative IHC scores (n = 6) of GTF2E2 in lung and liver of mice. D. Survival curves of the indicated metastasis animal groups. *p < 0.05, **p < 0.01, ***p < 0.001, ****p < 0.0001 vs. control. n=10/group.

**
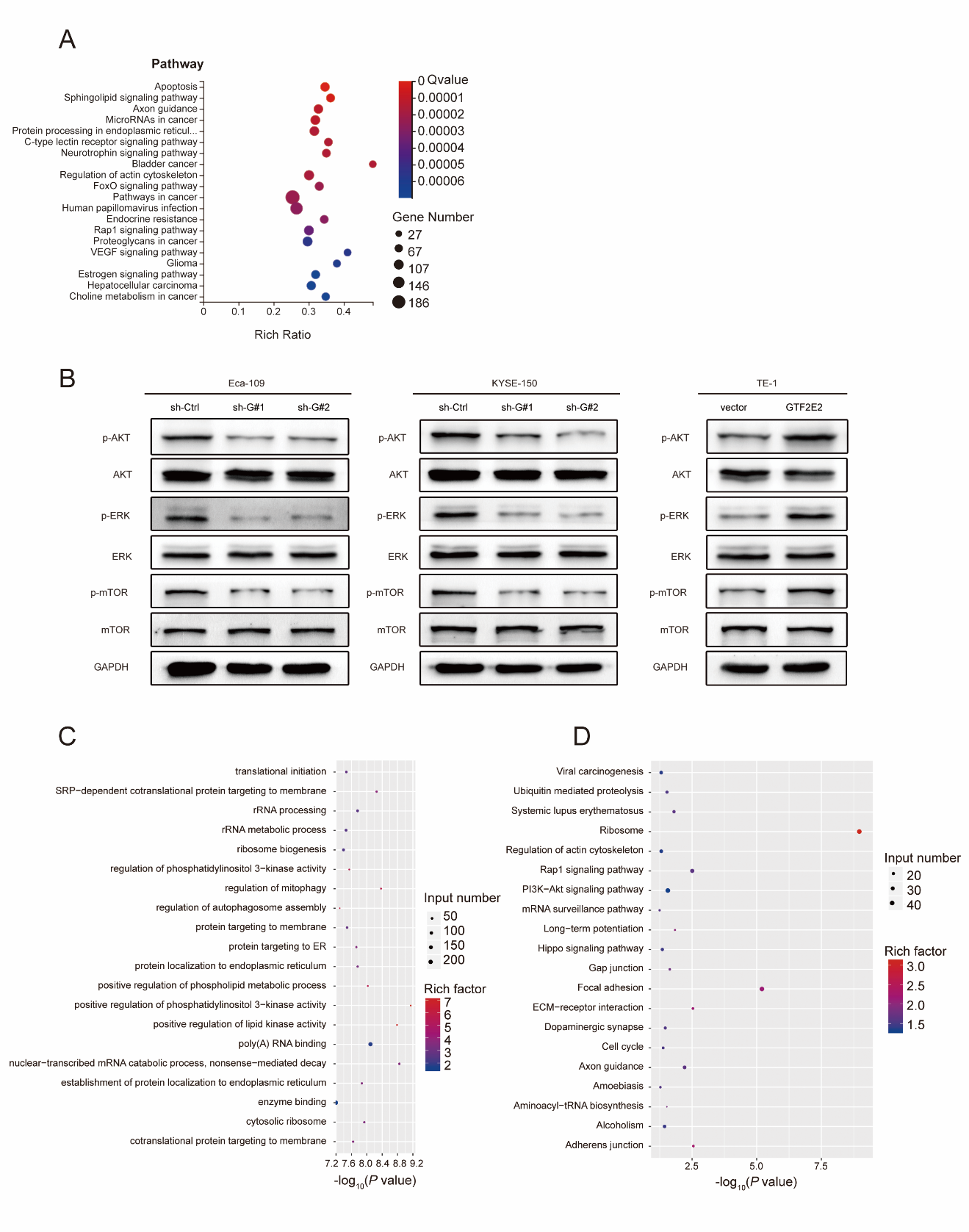
Figure S3. GO and KEGG analysis of RNA-seq and ChIP-seq results.** A. KEGG enrichment of the significant pathways of differentially expressed genes. B. The phosphorylation level of AKT, ERK and mTOR were evaluated by western blot in the indicated ESCC cells. C. GO enrichment of the biological process of peak-related genes. D. KEGG enrichment of the crucial pathways of peak-related genes.

**
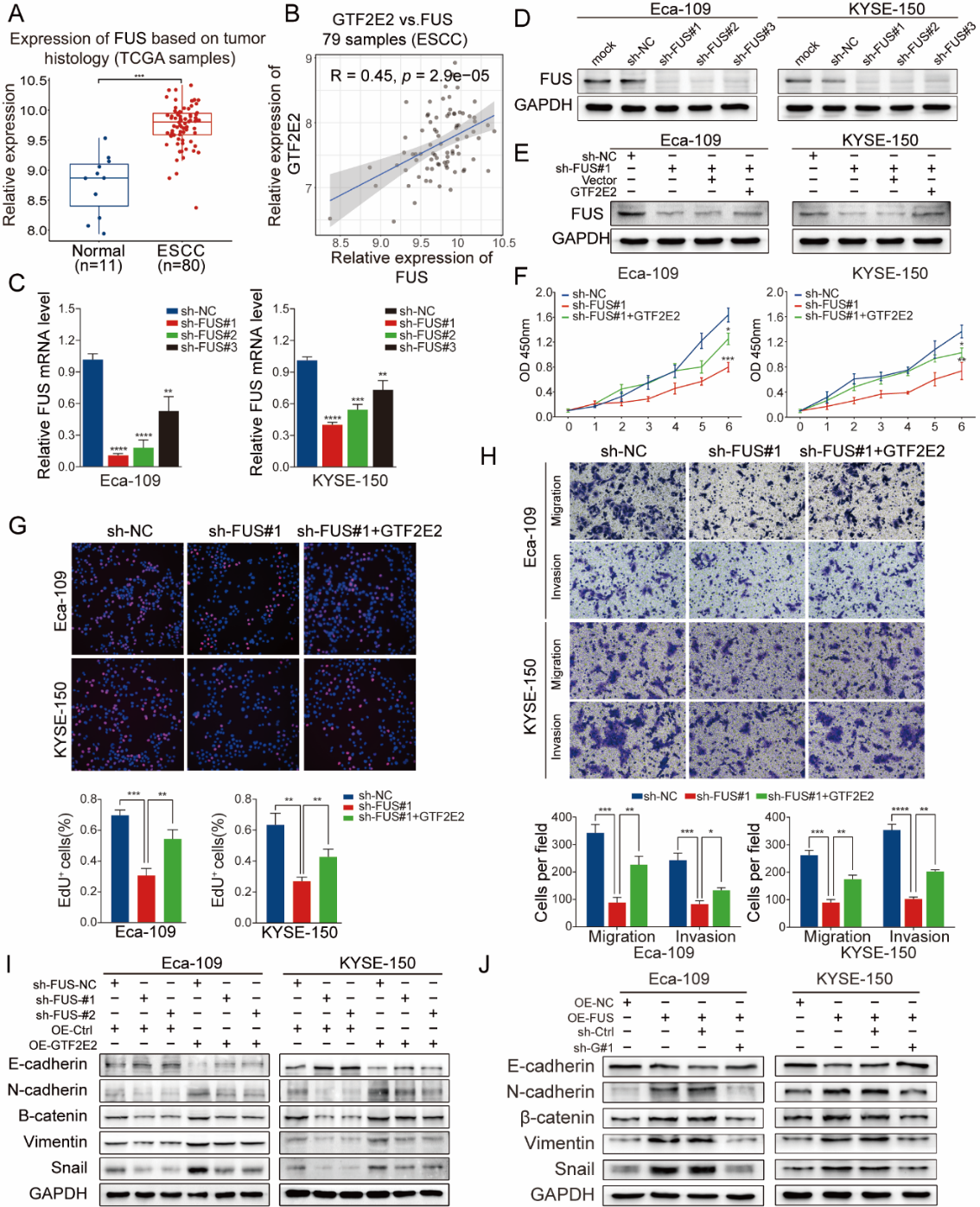
Figure S4. FUS mediates the GTF2E2-induced progression in ESCC cells *in vitro*.** A. Comparison of the FUS mRNA level between ESCC and normal tissues in TCGA database. B. The correlation analysis of FUS and GTF2E2 expression in TCGA ESCC samples. C-D. RT-PCR and western blot analysis of FUS knockdown efficiency in the indicated cells. GAPDH was used as a loading control. E. FUS-downregulating lentivirus was transfected into GTF2E2-upregulated ESCC cells, and FUS expression was confirmed by western blot. F-G. CCK8 and EdU assays were performed to determine the proliferation impact of ESCCs treated with GTF2E2 and FUS or negative control. Quantification of percentages of Edu+ cells. H. Transwell analysis showed the migration and metastasis capacity of ESCC cells co-transfected with GTF2E2 and FUS or negative control. Quantification of migrated and invaded cells. I-J. In western blot analysis, FUS downregulation or upregulation restored the changes in EMT protein markers induced by GTF2E2 overexpression or knockdown. *p < 0.05, **p < 0.01, ***p < 0.001, ****p < 0.0001 vs. control. n=3.

**
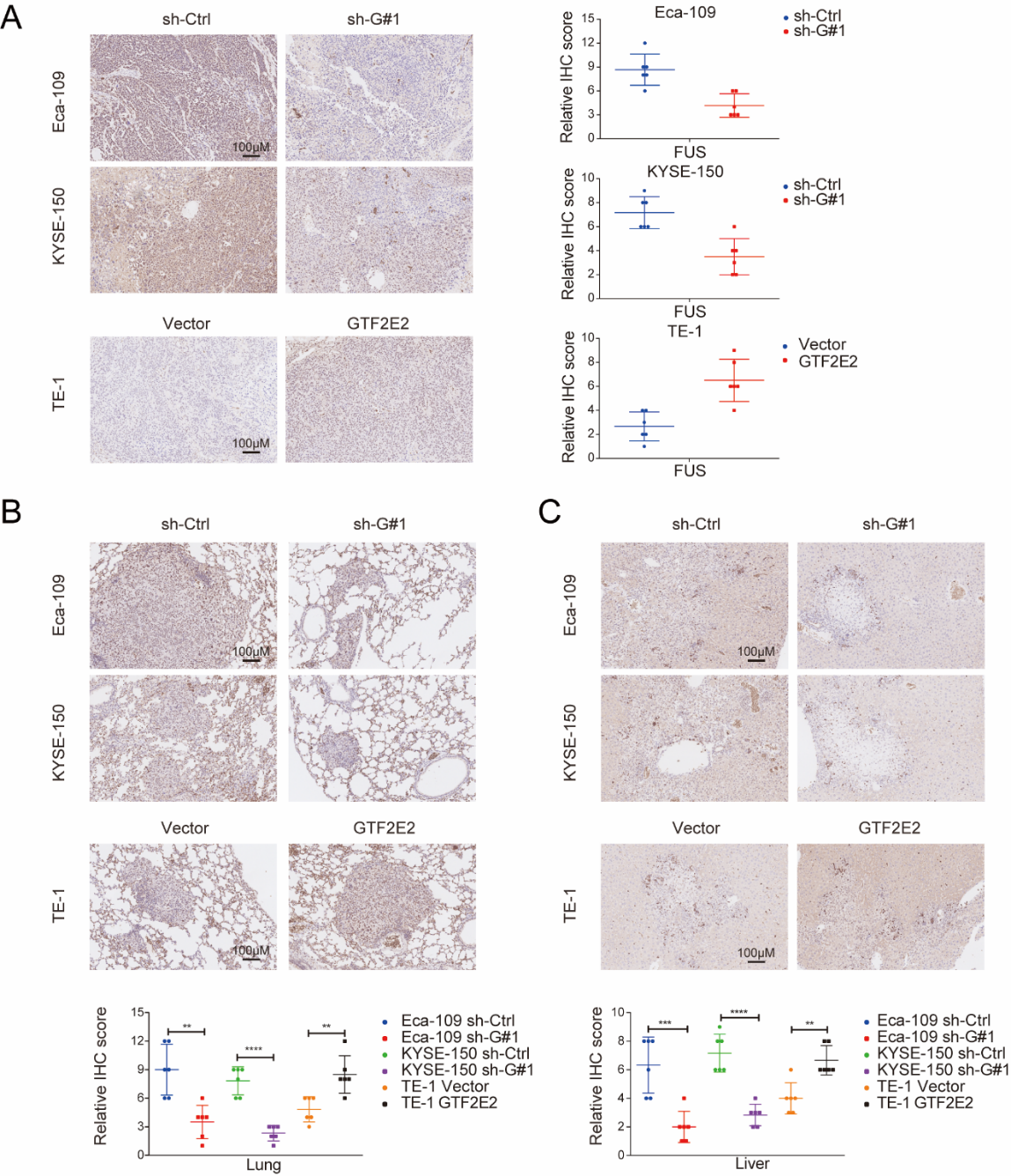
Figure S5. FUS mediates the GTF2E2-induced progression in ESCC cells *in vivo*.**

A. Representative images of IHC staining and the relative IHC scores (n = 6) of FUS in ESCC tumor xenografts of mice. B-C. In IHC analysis, FUS expression was markedly downregulated in the lung (B) and liver (C) metastatic tumors in nude mice with the injection of sh-G#1 cells from tail veins compared with control metastatic tumors in mice with sh-Ctrl cells. The opposite results were observed in TE-1 cells with GTF2E2 upregulation. *p < 0.05, **p < 0.01, ***p < 0.001, ****p < 0.0001 vs. control. n=10/group.

**
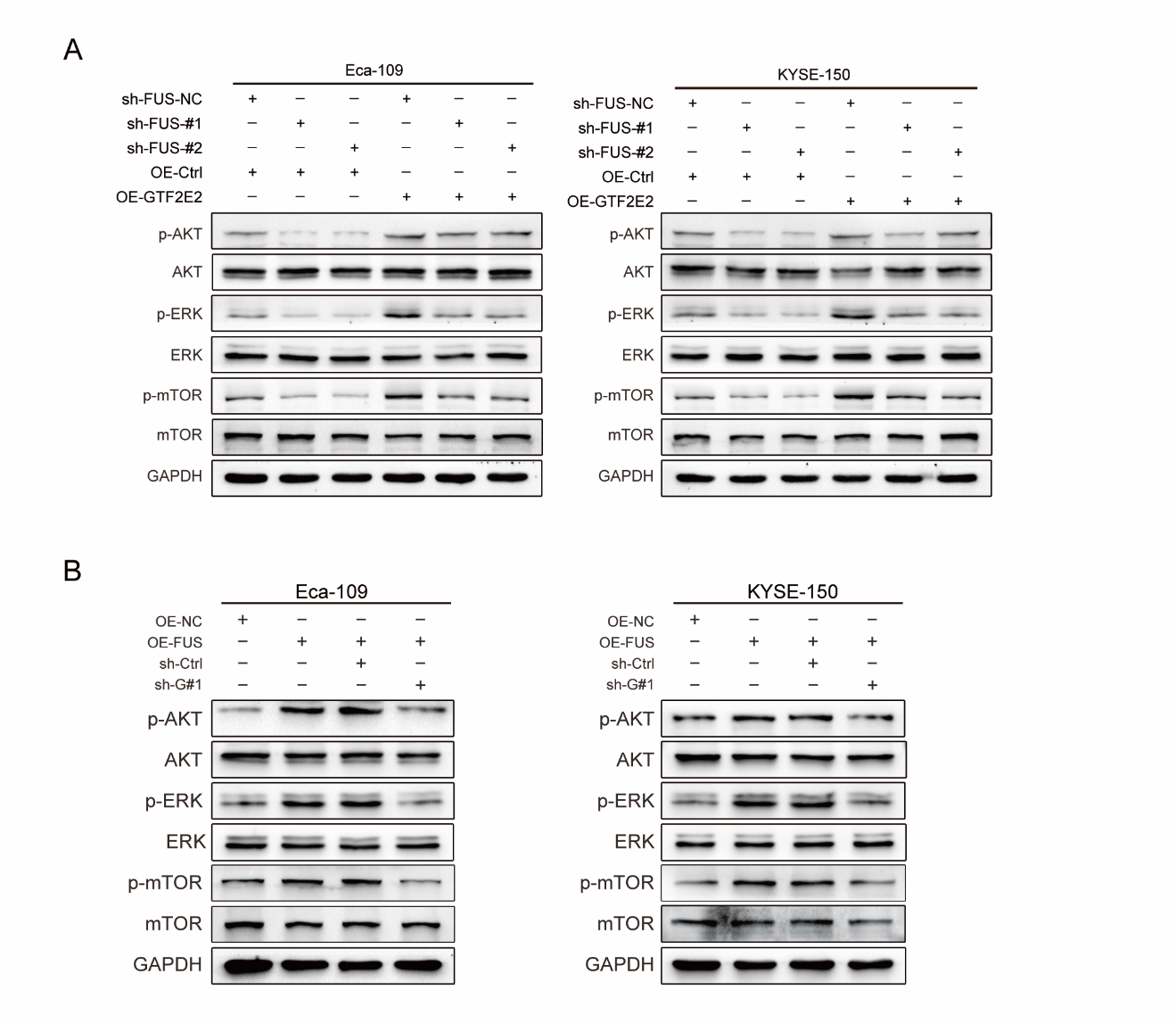
Figure S6. GTF2E2 plays an oncogenic role in ESCC via activation of the AKT/ERK/mTOR pathway.** A-B. In Eca-109 and KYSE-150 cells, western blot analysis showed that FUS downregulation or upregulation restored the changes in the phosphorylation level of AKT, ERK and mTOR induced by GTF2E2 overexpression or knockdown. *p < 0.05, **p < 0.01, ***p < 0.001, ****p < 0.0001 vs. control. n=3.
