# Supplementary material for: GTF2E2 is a novel biomarker for recurrence after surgery and promotes progression of esophageal squamous cell carcinoma via miR-139-5p/GTF2E2/FUS axis
Source: Oncogene. 2021 Dec 2;41(6):782–96. doi: 10.1038/s41388-021-02122-8 (PMC8816730; doi:10.1038/s41388-021-02122-8)
Supplement: Supplementary file 2 — Table S2 [file 41388_2021_2122_MOESM2_ESM.docx]

Table S2. The clinic-pathological characteristics and GTF2E2 scoring in postoperative recurrence ESCC specimens

| Characteristic | All (n=141) | Early recurrence  (n=35) | Late recurrence  (n=106) | P-value |
| --- | --- | --- | --- | --- |
| Median age (range) | 62 (46-79) | 59 (46-72) | 62 (51-79) | 0.404 |
| Age - N. (%) |  |  |  | 0.452 |
| <65 | 92 (65.2) | 21 (60.0) | 71 (67.0) |  |
| >=65 | 49 (34.8) | 14 (40.0) | 35 (33.0) |  |
| Gender |  |  |  | 0.038 |
| Male | 97 (68.8) | 29 (82.9) | 68 (64.2) |  |
| Female | 44 (31.2) | 6 (17.1) | 38 (35.8) |  |
| Performance status |  |  |  | 0.107 |
| 0 | 72（47.5） | 22 (62.9) | 50 (47.2) |  |
| 1 | 69（52.5） | 13 (37.1) | 56 (52.8) |  |
| Tumor location |  |  |  | 0.622 |
| Upper | 30 (21.3) | 8 (22.9) | 22 (20.8) |  |
| Middle | 54 (38.3) | 11 (31.4) | 43 （40.5） |  |
| Lower | 57 (40.4) | 16 (45.7) | 41 （38.7） |  |
| T stage |  |  |  | 0.154 |
| T1/T2 | 44 (31.2) | 7 (20) | 37 (34.9) |  |
| T3/T4 | 97 (68.8) | 28 (80) | 69 (65.1) |  |
| N stage |  |  |  | 0.562 |
| N0 | 19 (13.5) | 5 (14.3) | 14 (13.2) |  |
| N1-3 | 122 (86.5) | 30 (85.7) | 92 (86.8) |  |
| Clinical Stage |  |  |  | 0.213 |
| IB/IC | 10 (7.1) | 3 (8.5) | 7 (6.6) |  |
| IIA/IIB | 13 (9.2) | 2 (5.7) | 11 (10.4) |  |
| IIIA/IIIB/IIIC | 76 (53.9) | 15 (42.9) | 61 (57.5) |  |
| IVA | 42 (29.8) | 15 (42.9) | 27 (25.5) |  |
| G Stage |  |  |  | 0.246 |
| G1 | 21 (14.9) | 8 (22.9) | 13 (12.3) |  |
| G2 | 55 (39.0) | 14 (40.0) | 41 (38.7) |  |
| G3 | 65 (46.1) | 13 (37.1) | 52 (49.0) |  |
| Adjuvant therapy |  |  |  | 0.812 |
| None | 37 (26.3) | 7 (20.0) | 30 (28.3) |  |
| Cisplatin+docetaxel-RT | 66 (46.8) | 18 (51.4) | 48 (45.3) |  |
| Cisplatin+docetaxel | 11 (7.8) | 3 (8.6) | 8 (7.5) |  |
| RT (radiotherapy) | 27 (19.1) | 7 (20.0) | 20 (18.9) |  |
| GTF2E2 scoring | 3 (2-6) | 8 (6-9) | 2.5 (1-4) | ＜0.0001 |

P-value < 0.05 is significant.
